# Reliability and validity of two frequently used self-administered physical activity questionnaires in adolescents

**DOI:** 10.1186/1471-2288-8-47

**Published:** 2008-07-15

**Authors:** Vegar Rangul, Turid Lingaas Holmen, Nanna Kurtze, Koenraad Cuypers, Kristian Midthjell

**Affiliations:** 1 Nord-Trøndelag University College, Faculty of Health Science, Levanger, Norway; 2 HUNT Research Centre, Faculty of Medicine, Department of Public Health and General Practice, Norwegian University of Science and Technology, Verdal, Norway; 3 SINTEF Health Research, Department of Living Conditions and Service Delivery, Oslo, Norway

## Abstract

**Background:**

To create and find accurate and reliable instruments for the measurement of physical activity has been a challenge in epidemiological studies. We investigated the reliability and validity of two different physical activity questionnaires in 71 adolescents aged 13–18 years; the WHO, Health Behaviour in Schoolchildren (HBSC) questionnaire, and the International Physical Activity Questionnaire (IPAQ, short version).

**Methods:**

The questionnaires were administered twice (8–12 days apart) to measure reliability. Validity was assessed by comparing answers from the questionnaires with a cardiorespiratory fitness test (VO_2peak_) and seven days activity monitoring with the ActiReg, an instrument measuring physical activity level (PAL) and total energy expenditure (TEE).

**Results:**

Intraclass correlation coefficients for reliability for the WHO HBSC questionnaire were 0.71 for frequency and 0.73 for duration. For the frequency question, there was a significant difference between genders; 0.87 for girls and 0.59 for boys (*p *< 0.05). The intraclass correlation coefficients the IPAQ varied between 0.10 and 0.62 for the reliability. Spearman correlation coefficients for validity for both the WHO HBSC questionnaire and the IPAQ (recoded into low, moderate and high activity) measured against VO_2peak _were fair, ranging between 0.29 – 0.39. The WHO HBSC questionnaire measured against VO_2peak _for girls were acceptable, ranging between 0.30 – 0.55. Both questionnaires, except the walking question in IPAQ, showed a low correlation with PAL and TEE, ranging between 0.01 and 0.29.

**Conclusion:**

These data indicate that the WHO HBSC questionnaire had substantial reliability and were acceptable instrument for measuring cardiorespiratory fitness, especially among girls. None of the questionnaires however seemed to be a valid instrument for measuring physical activity compared to TEE and PAL in adolescents.

## Background

Physical activity during adolescence is positively related to physical fitness and health both in adulthood and later life [1,2]. However, physical activity is not synonymous with physical fitness. Physical activity is defined as any body movement produced by skeletal muscles resulting in a substantial increase in energy expenditure [3], while physical fitness is a set of attributes related to people's ability to perform physical work [4]. The cardiorespiratory component of physical fitness is related to the ability to perform dynamic large muscle mass work at moderate to high physical intensity over a prolonged period. This is important from a health point of view [3]. Physical fitness measured as cardiorespiratory fitness (maximal oxygen uptake) is positively related to improved health in general and to prevention of cardiovascular diseases in particular [5,6]. Recent studies show that the dose-response gradient for various health outcomes is steeper across categories of cardiorespiratory fitness than across groups with different levels of physical activity [7]. The greatest improvements in health status have been found when people who are sedentary become physically active. Church et al. [8] found a graded dose-response change in fitness across different levels of physical activity, and even exercise at only 50% of the physical activity recommendations provided some improvement on fitness. This promotes the understanding and importance of frequent physical activity at any level, thus even low physical activity is beneficial [9].

Representative data are essential in order to assess and monitor physical activity and physical fitness in a population and to study time trends. In epidemiological studies the use of self-reported questionnaires is often the only feasible method [10]. Self-reported questionnaires assessing vigorous physical activity have shown acceptable reliability and validity for adults [11,12]. The challenge is to get valid data for moderate and low physical activity [13]. To assess trends in the population a standardised questionnaire is strongly recommended [14]. Finding an accurate and reliable measurement of physical activity for children and adolescents is especially challenging because this group most often lacks a precise understanding of concepts like physical activity, exercise, sport and fitness [4]. In addition, these concepts are often not precisely defined in questionnaires. It is therefore of particular importance to study the validity and reliability of questionnaires aiming at measuring physical activity among adolescents.

One frequently used questionnaire for children and adolescents is the World Health Organization Health Behaviour in Schoolchildren (WHO HBSC) Survey Questionnaire [15]. To our knowledge only one study, conducted among Australian students, has validated the questions about physical activity in the WHO HBSC survey [16]. The International Physical Activity Questionnaire (IPAQ) is a physical activity questionnaire designed by a multinational working group as a common instrument for epidemiological studies [17]. Long and short versions of the IPAQ are available. It is designed for adults aged 15–65 years [18], but has only been validated for those 18 years and older [17,19,20]. The questionnaire has been used to monitor physical activity among people aged 15–78 years [21].

The purpose of this study was to investigate the reliability and validity of the physical activity questions from the WHO HBSC questionnaire and from the IPAQ (short version) among adolescents aged 13–18 years. Comparisons were done with objective measures of both physical activity and physical fitness.

## Methods

### Participants

The study population was recruited from two municipalities in Nord-Trøndelag County, Norway. The participants, aged 13–18 years old, were identified and randomly selected from four different schools in the included municipalities. An invitation was distributed by the teachers at the schools. Each participant received an information folder and signed a written consent. The parents of participants below 16 years also signed the consent.

Participation in the study was voluntary. A total of 200 adolescents were invited, 71 participated. With an estimated effect size of 0.5 and power of 80% (two-tailed alpha = .05), between the scores from the physical activity questionnaires and the objective measures, the study needed a sample of 58 participants

### Instruments

#### Physical activity questionnaires

The WHO HBSC Physical Activity Questionnaire has recently been used in two extensive studies in Norway; The Young-HUNT Study (adolescents 13–19 years, the Youth Part of the second Nord-Trøndelag Health Study, HUNT 2), and in the Health Behaviour in Schoolchildren study (HEVAS/HBSC) [15,22]. The questionnaire records the responder's physical activity level in sports and exercise by asking the adolescent to report the frequency and total amount of time spent exercising vigorously outside school hours. The frequency question was: "Outside school hours: How often do you usually exercise in your free time so much that you get out of breath or sweat?". The frequency question had eight response alternatives: "every day", "4–6 days a week", "2–3 days a week", "one day a week", "not every week, but at least once every 14^th ^day", "not every 14^th ^day, but at least once a month", "less than once a month" and "never". The duration question was: "Outside school hours: How many hours do you usually exercise in your free time, so much that you get out of breath or sweat?". The duration question had six response alternatives: "7 hours per week or more", "about 4–6 hours a week", "about 2–3 hours a week", "about one hour a week", "about half an hour a week" and "none". Answers were recoded into three categories of physical activity for both frequency and duration. "Low activity" represent "one day a week or less" or "one hour a week or less"; "moderate activity" represent "2–3 days a week" or "2–3 hours a week"; "high activity" represent "four days a week or more" or "four hour a week or more" (Table 1).

**Table 1:** Classification of physical activity by three categories

Category	Description			
	
	ActiReg	WHO HBSC frequency question	WHO HBSC duration question	IPAQ
Low activity	METs < 3	Exercise one day a week or less, so much that you get out of breath or sweat	One hour a week or less of exercise, so much that you get out of breath or sweat	Individuals who do not meet the criteria for moderate-intensity and vigorous-intensity activity categories are considered inactive.

Moderate activity	METs 3–6	Exercise 2–3 days a week, so much that you get out of breath or sweat	2–3 hours a week of exercise, so much that you get out of breath or sweat	• 3 or more days pr. week of vigorous activity, at least 20 minutes per day OR
				• 5 or more days pr. week of moderate intensity activity or walking, at least 30 minutes per day OR
				• 5 or more days pr. week of any combination of walking, moderate-intensity or vigorous-intensity activities achieving a minimum of at least 600 MET-min/week.

High activity	METs > 6	Exercise four days or more a week, so much that you get out of breath or sweat	Four hours or more a week of exercise, so much that you get out of breath or sweat	• Vigorous-intensity activity on at least 3 days pr. week and accumulating at least 1500 MET-min/week OR
				• 7 or more days pr. week of any combination of walking, moderate-intensity or vigorous-intensity achieving a minimum of a least 3000 MET-min/week.

METs = Intensity of activity compared to resting energy expenditure

The International physical activity questionnaire (IPAQ), self-administered short version was designed for use among young and middle aged adults, 15–69 years old [18]. The questionnaire inquires activity during the last week. The questions focus on four activity types: "vigorous activity" periods for at least 10 min; "moderate activity" periods for at least 10 min, "walking" periods for at least 10 min and times spent "sitting" on weekdays. Frequency of activity is measured in days and duration in hours and minutes. Answers from the IPAQ were recoded in a categorical score, classified into three categories (Table 1); "low", "moderate" and "high" physical activity as defined by the IPAQ working group [18].

#### Maximal oxygen uptake (VO_2peak_)

A metabolic analyzer, Metamax II (Cortex Biophysic GmbH, Leipzig, Germany), was used for measuring VO_2peak_. The measurements were done in the participants' schools. The analyser recorded and displayed data every 10th second. The data collected were stored, using the program Cortex Metasoft. The Metamax II has been validated applying the Douglas bag technique as the criterion method [23].

The instrument has built-in sensors for O_2 _and CO_2_. It contains a barometer and a thermometer and measures the flow of the breathed air by means of a turbine flow meter attached to the breathing mask. Before each test started, the instrument was calibrated against ambient air and a commercial gas with known concentrations of O_2 _(16%) and CO_2 _(4%). The concentration of O_2 _and CO_2 _in room air was recorded, and the flow transducer was calibrated using a 3-L high-precision calibration syringe (Calibration syringe D, Sensor Medics, Yorba Linda, CA) before testing each participant.

#### ActiReg

The ActiReg (PreMed AS, Oslo, Norway) is an activity monitor recording both body position and movement, contrary to an accelerometer, which records body position only. The ActiReg has two pairs of position and motion sensors connected by cables to a battery-operated storage unit fixed to a waist belt. Each pair of sensors was attached by medical tape to the chest and to the front of the right thigh respectively. The ActiReg distinguishes between four body positions; standing, sitting, bent forward and lying down. Every second the combination between body position and movement is registered, and every 60 seconds activity factors are calculated. An especially designed computer program, the ActiCalc, processes the collected data. This program stores all specific data and calculates energy expenditure. Description and validation of the ActiReg was published by Hustvedt et al. [24]. The ActiReg has been used to validate energy intake estimated from pre-coded food diaries in adolescents [25].

### Measurements

#### Anthropometric measures

Height and weight were measured with light clothes and without shoes in all participants. Height was me asured to the nearest 0.5 cm by a calibrated wall-mounted measuring instrument, while body weight was measured to the nearest 0.1 kg using a calibrated laboratory scale. Body mass index (BMI) was calculated as weight divided by height squared (kg·m^-2^).

#### Physical fitness

Physical fitness (cardiorespiratory fitness as VO_2peak_) was measured using a treadmill, applying the Oslo protocol, designed for children and adolescents [26]. The speed and incline were increased every second minute, one factor at the time. The starting level was speed at 5 km/h and an incline at 1%. The main criterion for VO_2peak _was the lack of further increase in O_2 _uptake or exhaustion. Participants were instructed not to eat or smoke for at least 2 hours before the test, to avoid high physical activity efforts the last 12 hours before the test and to wear clothing and shoes appropriate for exercise.

#### Physical activity

The ActiReg measured physical activity during seven consecutive days. The energy expenditure for each day was added up, and total energy expenditure (TEE) and physical activity level (PAL) were calculated. PAL is defined as TEE divided by basal metabolic ratio [27]. The ActiReg calculated a metabolic equivalent (MET) value each minute, which expresses intensity of the activity compared to resting energy expenditure (1 MET = 3.5 ml O_2_·kg^-1^·min^-1 ^or 1 kcal·kg^-1^·h^-1^) [28]. MET values were categorised in low (METs < 3), moderate (METs 3–6) and high activity (METs > 6) (Table 1). Basal metabolic rate was calculated using the FAO/WHO equation [29].

### Study design

#### Reliability

The reliability was evaluated applying a test-retest design. The questionnaires were completed a first time before taking the objective measurements and the second time, 8–12 days later.

#### Validity criteria

Criterion validity was assessed comparing the self-reported physical activity questions in the WHO HBSC and the IPAQ with physical fitness (cardiorespiratory fitness, VO_2peak_) and physical activity measured by the ActiReg. Cardiorespiratory fitness reflects the ability to transport and utilised oxygen during prolonged, strenuous physical activity. Physical activity was measured in total energy expenditure (TEE) and physical activity level (PAL) for seven days.

#### Ethics

The study followed the principles outlined in the Helsinki Declaration. It was approved by The Norwegian Data Inspectorate Board and recommended by The Regional Committee for Ethics in Medical Research.

#### Data analysis

SPSS Inc., Chicago IL, version 14.1 was used for all analyses. The statistical analyses were performed for the total group and stratified by gender and age. To evaluate reliability, we calculated single measure intraclass correlation coefficients (ICC). A 95% confidence interval (CI) was used to describe the variety/difference in the ICCs.

To assess the validity of the physical activity questionnaires we used Spearman rank correlation between the questionnaires and the objective measures (VO_2peak_, TEE and PAL). In the validity analyses, we used the answers from the first assessment for the WHO HBSC questions. For the IPAQ we used the answers from the second assessment, because the questions asked for activity the last seven days.

## Results

### Subject characteristics

Seventy-one participants, 56.3% girls, completed the questionnaire and anthropometric measurements. Mean age was 14.9 years (girls 15.3 years, boys 14.4 years) (Table 2). Sixty-seven participants completed the VO_2peak _measures (30 boys and 37 girls), while 62 (26 boys and 36 girls) completed all parts of the study.

**Table 2:** Physical characteristics of participants stratified by gender and age

Characteristic	All (*n *= 71)	Girls (*n *= 40)	Boys (*n *= 31)	13–15 years (*n *= 42)	16–18 years (*n *= 29)
Age (year)	14.9 (1.64)	15.3 (1.65)	14.35 (1.50)	13.64 (0.62)	16.66 (0.77)
Height (cm)	166 (8.37)	163.6 (6.49)	169.2 (9.53)	164.9 (6.93)	167.6 (10.08)
Weight (kg)	57.0 (11.33)	56.0 (9.88)	58.4 (13.08)	54.7 (9.76)	60.4 (12.76)
Body mass index (kg/cm^2^)	20.5 (2.86)	20.8 (2.81)	20.2 (2.94)	20.0 (2.76)	21.3 (2.89)

The values are presented by Means with Standard deviations in brackets

Boys had a significantly higher VO_2peak _compared to girls (Table 3), but there was no significant difference in VO_2peak _between age groups. The PAL values for seven days differed significantly between age groups. Adolescents 13–15 year olds were more physically active than the 16–18 year olds. Internally in category "METs < 3", boys were physically active for fewer minutes than to girls. The age group 13–15 year had significantly fewer minutes registered at "METs < 3" compared to the 16–18 year olds, while in the "METs 3–6" (minutes) the 13–15 year olds had significantly more minutes registered than age group 16–18. There were no significant age and gender differences concerning answers on physical activity in any of the questionnaires (Table 3).

**Table 3:** Physical fitness and physical activity stratified by gender and age

Measurements	All (*n *= 71)	Girls (*n *= 40)	Boys (*n *= 31)	13–15 years (*n *= 42)	16–18 years (*n *= 29)
**Physical fitness**					

VO_2peak _(l·min^-1^)	3.04 (0.77)	2.73 (0.58)*	3.44 (0.81)	2.93 (0.60)	3.24 (0.99)
VO_2peak _(ml·kg^-1^·min^-1^)	52.54 (8.12)	48.06 (6.21)*	58.06 (6.73)	53.14 (7.66)	51.53 (8.92)

**Physical activity**					

Actireg (PAL for 7 days)	1.70 (0.24)	1.66 (0.22)	1.77 (0.26)	1.75 (0.28)^#^	1.63 (0.14)
Actireg (TEE for 7 days)	59.39 (8.65)	57.72 (8.51)	61.70 (8.47)	59.97 (9.93)	58.47 (6.20)
ActiReg (min at METs < 3 for 7 days)	8,954 (441)	9,083 (314) *	8,775 (528)	8,850 (476) ^#^	9,118 (325)
ActiReg (min at METs 3–6 for 7 days)	845 (313)	776 (207)	942 (402)	953 (328) ^#^	675 (192)
ActiReg (min at METs > 6 for 7 days)	256 (210)	219 (180)	308 (240)	277 (249)	224 (122)

**Questionnaires**					

WHO HBSC questionnaire					
Frequency (days per week)	3.80 (1.77)	3.61 (1.57)	3.97 (2.01)	3.64 (1.81)	3.95 (1.73)
Duration (hours per week)	4.10 (1.29)	3.81 (2.15)	4.48 (2.36)	4.03 (2.23)	4.21 (2.32)
IPAQ					
Vigorous activity (days/week)	2.76 (1.84)	2.85 (1.78)	2.65 (1.94)	2.81 (1.70)	2.69 (2.06)
Vigorous activity (min/day)	73 (43)	71 (39)	74 (47)	78 (51)	65 (28)
Moderate activity (days/week)	2.89 (2.18)	2.93 (2.10)	2.84 (2.34)	2.98 (2.17)	2.76 (2.23)
Moderate activity (min/day)	65 (42)	65 (42)	66 (44)	71 (40)	61 (44)
Walking (days/week)	4.39 (2.19)	4.26 (2.08)	4.57 (2.34)	4.70 (2.26)	3.97 (2.04)
Walking (min/day)	43 (53)	44 (55)	40 (50)	48 (52)	36 (53)
Sitting (min/day)	374 (196)	414 (209)	327 (171)	289 (177)	484 (164)

The values are presented in means with standard deviations in brackets

PAL = Average physical activity level for 7 days (PAL = total energy expenditure/basal metabolic rate)

TEE = Total energy expenditure in mega joule

METs = Intensity of activity compared to resting energy expenditure

* Significant difference between genders (*p *≤ 0.01)

^# ^Significant difference between age groups (*p *≤ 0.05)

### Reliability

According to Landis and Koch divisions of agreement [30], the WHO HBSC questionnaire indicated a substantial overall reliability (frequency *r *= 0.73 and duration *r *= 0.71) (Table 4). Significant differences were found between girls and boys on the WHO HBSC frequency question (*r *= 0.87 and *r *= 0.59 respectively), and between age groups on the duration question (13–15 years *r *= 0.62 and 16–18 years *r *= 0.85).

**Table 4:** Test-retest reliability based on intraclass correlation coefficients (ICC) for the WHO HBSC questionnaire and the IPAQ

Questionnaire	All (*n *= 71)		Girls (*n *= 40)		Boys (*n *= 31)		13–15 years (*n *= 42)		16–18 years (*n *= 29)	
					
	ICC	95% CI	ICC	95% CI	ICC	95% CI	ICC	95% CI	ICC	95% CI
**WHO HBSC questionnaire**										

Frequency	0.73	0.60–0.82	0.87*	0.77–0.93	0.59	0.31–0.78	0.71	0.53–0.83	0.76	0.55–0.88
Duration	0.71	0.57–0.81	0.76	0.58–0.86	0.66	0.40–0.83	0.62^#^	0.39–0.78	0.85	0.70–0.93

**IPAQ**										

Vigorous activity (days/week)	0.54	0.34–0.69	0.55	0.29–0.73	0.53	0.22–0.75	0.46	0.18–0.67	0.65	0.37–0.82
Vigorous activity (min/day)	0.30	-0.07–0.56	0.57	0.20–0.80	0.24	-0.21–0.62	0.46	0.05–0.74	0.23	-0.23–0.61
Moderate activity (days/week)	0.55	0.36–0.70	0.58	0.33–0.75	0.53	0.21–0.75	0.57	0.32–0.74	0.53	0.20–0.75
Moderate activity (min/day)	0.34	0.22–0.60	0.36	-0.06–0.67	0.33	-0.20–0.72	0.67	0.25–0.88	0.21	-0.21–0.57
Walking (days/week)	0.62	0.45–0.75	0.53*	0.27–0.72	0.77	0.56–0.89	0.81^#^	0.67–0.90	0.37	0.01–0.65
Walking (min/day)	0.10	-0.10–0.39	0.06	-0.33–0.44	0.11	-0.35–0.54	0.11	-0.30–0.49	0.07	-0.36–0.49
Sitting (min/per day)	0.27	-0.50–0.54	0.18	-0.22–0.54	0.43	-0.09–0.77	0.32	-0.13–0.67	0.03	-0.41–0.46

ICC = Single measure intraclass correlation coefficient

* Significant difference between genders (*p *< 0.05)

^# ^Significant difference between age groups (*p *< 0.05)

The overall reliability of the IPAQ questionnaire varied for the different physical activity categories. The lowest correlation was found for walking (minutes per day) (*r *= 0.10), while the highest correlation was found for walking (days per week) (*r *= 0.62). The IPAQ walking (days) question showed statistically significant difference between genders (girls *r *= 0.53 and boys *r *= 0.77) and age groups (13–15 years *r *= 0.81 and 16–18 years *r *= 0.37) (Table 4).

### Validity

For the total population, a statistically significant correlation was found between VO_2peak _and the questions on both frequency (*r *= 0.39) and duration (*r *= 0.33) in the WHO HBSC questionnaire (Table 5). The correlation was also significant when the answers were divided into three categories (Table 5). Girls had a higher correlation between the WHO HBSC questionnaire and VO_2peak _(*r *varied between 0.41 and 0.55) compared to boys (*r *varied between 0.21 and 0.31), and correlations were statistically significant in girls only.

**Table 5:** Spearman rank-correlation coefficients for the WHO HBSC questionnaire and the IPAQ against VO_2peak_, TEE and PAL to assess validity

Questionnaire	VO_2peak _(l·min^-1^)			TEE			PAL		
			
	All (*n *= 67)	Girls (*n *= 37)	Boys (*n *= 30)	All (*n *= 62)	Girls (*n *= 36)	Boys (*n *= 26)	All (*n *= 62)	Girls (*n *= 36)	Boys (*n *= 26)
**WHO HBSC**									

Frequency	0.39**	0.55**	0.31	0.20	0.25	0.08	0.02	0.01	-0.07
Duration	0.33**	0.41*	0.21	0.23	0.21	0.24	0.01	-0.1	0.07
Frequency, 3 categories	0.36**	0.53**	0.31	0.22	0.28	0.11	0.02	0.05	-0.06
Duration, 3 categories	0.29*	0.30	0.31	0.22	0.23	0.25	-0.02	-0.08	0.03

**IPAQ**									

Vigorous activity (days/week)	0.26*	0.37*	0.02	0.19	0.20	0.10	0.09	0.03	0.05
Vigorous activity (min/day)	-0.32*	-0,27	-0.31	-0.14	-0.02	-0.29	-0.08	0.12	-0.08
Moderate activity (days/week)	-0.03	0.11	0.04	0.07	0.04	0.18	0.05	-0.02	0.14
Moderate activity (min/day)	0.13	0.02	-0.17	0.01	-0.17	0.25	0.01	-0.09	0.10
Walking (days/week)	0.12	0.19	-0.12	0.15	0.11	0.22	0.13	0.05	0.25
Walking (min/day)	-0.14	-0.41*	0.20	0.24	0.15	0.38	0.43**	0.28	0.61**
Sitting (min/day)	0.18	0.33	0.30	-0.04	0.54**	-0.42	-0.29	0.25	-0.68**
3 categories	0.32**	0.43**	0.18	0.09	011	-0.02	-0.03	-0.12	-0.05

TEE = Total energy expenditure for 7 days.

PAL = Average physical activity level for 7 days.

* *p *< 0.05;

** *p *< 0.01

3 categories = Classification of physical activity in three levels; "low", "moderate" and "high" activity

The correlation coefficients of the WHO HBSC questions measured against the TEE and PAL was low (Table 5).

Vigorous activity (days per week) measured in the IPAQ and classified into three categories, was significantly correlated with VO_2peak _(Table 5). Vigorous activity (minutes per day) and walking (minutes per day) in the IPAQ correlated negatively with VO_2peak_, indicating that more minutes of both vigorous activity and walking was associated with a lower VO_2peak_. There was, however, a significant correlation between the IPAQ expressed as walking (minutes per day) and VO_2peak _for girls (*r *= -0.41).

The correlation coefficient between the IPAQ questions and PAL was significant for walking (minutes per day) including all (*r *= 0.43) and for boys when split by gender (*r *= 0.61). The IPAQ question on sitting (minutes per day) showed a significant negative correlation with PAL for boys (*r *= -0.68) and was significantly correlated with TEE in girls (*r *= 0.54). The other associations between the IPAQ questions and the ActiReg measures had a low correlation and were not significant (Table 5).

## Discussion

The WHO HBSC physical activity questionnaire had a substantial reliability concerning frequency as well as duration of activity, and validity expressed as the spearman correlation coefficient between the answers and physical fitness (VO_2peak_). The IPAQ question on vigorous activity (days per week) and recoded into three categories showed a fair correlation with physical fitness (VO_2peak_). The other questions had a low validity against VO_2peak_. Measured against TEE and PAL (ActiReg, 7-day records), validity for both questionnaires was low.

### Reliability

In general the reliability of WHO HBSC questionnaire was comparable to a study among Australian high school students [16]. An interesting observation in our study is that the WHO HBSC questionnaire tended to be more reliable for girls. A reliability study by Treuth et al. [31] found no gender difference in the Fels physical activity questionnaire for children. Few studies have however, focused on possible gender differences. The gender differences in our study could be due to the fact that girls tend to be more precise in their answers. Girls probably are less competitive than boys concerning physical activity, and thus they may be more "honest" in their answers. Girls value different things and they do not need to emphasise themselves as very physically active. This may strengthen the reliability patterns for girls.

Our results also revealed a difference between age groups. The WHO HBSC questionnaire was more reliable for the oldest group. This is similar to what Treuth et al. found in their study [31]. The lower reliability in the 13–15 year old could be due to a failure to interpret the questions correctly. Those 16–18 years probably had a better understanding of its contents, explaining the higher correlation in the oldest group. The reliability of the IPAQ was lower than that of the WHO HBSC questionnaire. This could be explained by the less structured format (open-ended questions) in the IPAQ. The lower test-retest reliability of the IPAQ could also be related to the reference period, because the questionnaire focuses on the last seven days, while physical activity may change considerably from one week to the next.

Concerning the IPAQ there were only minor differences between genders, except for the question about walking/days, where boys had a higher reliability than girls. The same was shown in the IPAQ, 12-country reliability and validity study among adults [17].

### Validity

In our study, we used two objective methods to validate the questionnaires; physical activity (TEE and PAL) and physical fitness (VO_2peak_). Physical activity is difficult to measure. Validating self-reported physical activity by questionnaire is therefore a great challenge. Different methods have been applied as validation criteria; accelerometer, pedometer, recall-logs, heart-rate monitoring and different energy expenditure methods [32]. The doubly labelled water method (DLW), indirect calorimetry and direct observations are the most reliable and valid measurements. The DLW method has drawbacks like financial costs and limitations due to the laboratory test situation [32]. Accelerometers have become increasingly popular as measurement tools for physical activity. However, inaccuracies, especially related to underestimation and inconsistency in the definition of what constitutes light, moderate, and vigorous activity have been reported [33-35]. The ActiReg, used in this study, has been validated against DLW and indirect calorimetry. Hustvedt et al. [24] found good agreement in moderate activity groups (moderately activity from 38 to 104 min per day) with a mean PAL of 1.70, which is comparable to our mean and therefore support the representativeness of our sample. The ActiReg has some limitations for PAL above 1.70. Applied on a population with low PAL (patients with chronic obstructive pulmonary disease), the ActiReg is found to be a valid tool to assess energy expenditure and distinguish between both the low intensity activity range and moderate to high intensity activity range of physical activity [36]. ActiReg is also able to recognise activities such as sitting and different intensities of movements. We have analysed these separately, but these analyses showed no changes in results (data not shown)

Physical fitness has been related to total and cardiovascular mortality and heart disease. Therefore, cardiorespiratory fitness, measured as VO_2peak_, has been preferred as the validation criterion for physical fitness in the last decades, and is considered the gold standard in the assessment of exercise tolerance [37].

There was a significant correlation between the WHO HBSC questionnaire and VO_2peak _for all, except for boys when analysed stratified by gender. When each question in the WHO HBSC questionnaire was analysed separately, the frequency question had a higher correlation than the duration question. The same trend was evident when split by gender. A possible explanation for the differences in the dimensions (duration and frequency) is that the frequency question, which inquires days per week, estimated physical activity more precisely than the duration question, which requests hours per week of physical activity. Another explanation could be related to the fact that days per week are a rougher estimate than hours per week. The IPAQ had a low validity measured against VO_2peak_, except for the question about vigorous physical activity during the last 7 days for all. This corresponds to previous research showing that vigorous activity is easier to recall than light activity [38]. Craig et al. [17] reported a typical correlation coefficient for the IPAQ was 0.30 for validity. In our study, the IPAQ recoded in three categories had an acceptable correlation against VO_2peak _for all (0.32) as well as separately for girls when split on gender. Nevertheless, each question separately was not a valid measure of physical fitness. The IPAQ seemed to be an acceptable instrument when the questions were compiled. This is important because physical activity is most often recoded and classified using a scoring protocol in epidemiological studies.

The validity for single IPAQ questions within "moderate activity", "walking" (days per week) and "sitting" was poor. "Walking", expressed as minutes per day, was negatively correlated to VO_2peak_. This probably means that the girls reporting walking for small distances do not perform vigorous physical activity, and that those with a high intensity activity associated with high cardiorespiratory fitness are inclined not to report lower physical activity like walking. To improve cardiorespiratory physical fitness sedentary persons need an intensity 40 to 60% of maximal aerobic power, corresponds to being slightly out of breathing or sweating [39]. In general an exercise intensity above 80% to 90% of the individuals' maximal aerobic power (vigorous activity; MET > 6) is recommended to increase VO_2peak _[40].

The ActiReg registered all activity performed by the participants for seven consecutive days. It was surprising that only the IPAQ questions about walking and sitting expressed as minutes a day showed significantly negative correlations against the TEE and PAL. Adolescents who reported many walking minutes had a high PAL value. For the IPAQ measured as sitting (minutes per day), these findings indicated that the girls who reported many sitting minutes had a high TEE. In the boys, however, we found the opposite; namely that those who reported many sitting minutes had a lower TEE, which we would expect. The low correlations between IPAQ and TEE/PAL could be related to underreporting of vigorous and moderate activity. We also found this underreporting in the WHO HBSC questions and this could explain the low validity measured against the ActiReg. It is difficult to explain the lack of correlation between the questionnaires and the ActiReg. Based on our results one consideration is that the WHO HBSC questionnaire and the IPAQ have questions which are related to activities that increase cardiorespiratory fitness [15,19], and therefore correlate better with VO_2peak _than TEE and PAL. However, this large difference between the answers and ActiReg could also be caused by the difficulties in creating accurate questions, and this could be an indication that we should prefer objective methods to measure physical activity in youth [41]. Another explanation might be that our participants were younger than the age group for which the IPAQ was designed, and thus might not fully understand the questions. Recall bias in questionnaires, especially among adolescents, may influence the retrospective response. Active adolescents tend to overestimate physical activity, whereas obese adolescents underestimate physical activity [42]. These variations may result in weaker correlations, thus influencing the validity. Because regular physical activity over a long period leads to physical fitness, we would expect good correlation between answers in questionnaires on physical activity and both ActiReg and VO_2peak_.

VO_2peak _is a more stable measure than physical activity. Physical activity may change daily, and from one week to next week, while physical fitness does not change considerably in 2–3 weeks' time. A possible bias could be related to the reference period, and we therefore did not find correlations on the criterion physical activity measure.

In our study, the questionnaire answers tended to underestimate physical activity, compared to the ActiReg (Table 3). The individual variations and the underestimations could be the explanation on the low validity, compared with PAL and TEE, and illustrates the difficulty to capture the individual energy expenditure in questionnaires [43].

For an accurate validation, the strength of our study is the use of two objective measures to validate the questionnaires. The sample size in this study is an additional aspect. Our response rate of 35% is, however, rather low and might introduce a risk of an overrepresentation of those who are most physically active. Based on the participants' cardiorespiratory fitness and BMI, our population were, however quite comparable to those in other studies [44,45], including the Young-HUNT study, including 90% of the population 13–19 years of age in Nord-Trøndelag County (data not shown). This indicates a low selection bias of our population.

Our findings of higher correlations with VO_2peak _than TEE and PAL could be because adolescents report vigorous activity most precisely. Respondents with a high-energy expenditure may not necessarily have high VO_2peak_. Adolescents, who perform vigorous physical activity and thereby have a high VO_2peak_, may do little moderate activity and therefore have relatively lower total energy expenditure. Although physical activity and physical fitness are two different dimensions, they are linked and both are correlated to health and survival [13].

## Conclusion

The WHO HBSC questions seemed to be acceptable instruments to measure cardiorespiratory fitness for girls. The IPAQ (recoded into three categories) seemed to be a fair instrument but based on our results none of the questionnaires seemed to be a valid instrument for measuring physical activity among adolescents. In addition, the answers from girls were more reliable and valid than the answers from boys. Thus, validity and reliability of the WHO HBSC questionnaire were acceptable, while validity of the IPAQ was fair for girls. But, they may become better instruments if gender differences are taken into account and the distinction between assessing physical activity and physical fitness is made more precise. These issues should be addressed in the near future.

## Competing interests

The authors declare that they have no competing interests.

## Authors' contributions

All authors read and approved the manuscript. VR made a substantial contribution to the initial conception of the research reported in this study, designing this study, collecting data, analyzing, interpreting data and wrote the original manuscript. TLH made a substantial contribution to writing this paper and revising drafts for important contents. NK made a substantial contribution to designing this study and revising drafts for important contents. KC made a substantial contribution to collecting data and analyzing and interpreting data. KM made a substantial contribution to designing this study to answer the research questions, interpreting data and revising drafts for important intellectual contents.

## Pre-publication history

The pre-publication history for this paper can be accessed here:



## References

[B1] Biddle SJ, Gorely T, Stensel DJ (2004). Health-enhancing physical activity and sedentary behaviour in children and adolescents. J Sports Sci.

[B2] Blair SN, LaMonte MJ, Nichaman MZ (2004). The evolution of physical activity recommendations: how much is enough?. Am J Clin Nutr.

[B3] Boucard CH, Shepard RJ, C B, RJ S and T S (1994). Physical Activity, Fitness, and Health: The Model and Key Concepts.. Physical Activity, Fitness, and Health International Proceedings and Consensus Statement.

[B4] Caspersen CJ, Christenson GM, Powell KE (1985). Physical activity, exercise, and physical fitness: definitions and distinctions for health-related research.. Public Health Rep.

[B5] Blair SN, Kohl HW, Barlow CE, Paffenbarger RS, Gibbons LW, Macera CA (1995). Changes in Physical-Fitness and All-Cause Mortality - A Prospective-Study of Healthy and Unhealthy Men. JAMA.

[B6] Pate RR, Wang CY, Dowda M, Farrell SW, O'Neill JR (2006). Cardiorespiratory fitness levels among US youth 12 to 19 years of age - Findings from the 1999-2002 National Health and Nutrition Examination Survey. Arch Pediatr Adolesc Med.

[B7] Blair SN, Cheng Y, Holder JS (2001). Is physical activity or physical fitness more important in defining health benefits?. Med Sci Sports Exerc.

[B8] Church TS, Earnest CP, Skinner JS, Blair SN (2007). Effects of different doses of physical activity on cardiorespiratory fitness among sedentary, overweight or obese postmenopausal women with elevated blood pressure - A randomized controlled trial. JAMA.

[B9] Lee IM (2007). Dose-response relation between physical activity and fitness - Even a little is good; More is better. JAMA.

[B10] Kohl HW, Fulton JE, Caspersen CJ (2000). Assessment of physical activity among children and adolescents: A review and synthesis. Prev Med.

[B11] Kurtze N, Rangul V, Hustvedt BE, Flanders WD (2007). Reliability and validity of self-reported physical activity in the Nord-Trondelag Health Study (HUNT 2). Eur J Epidemiol.

[B12] Kurtze N, Rangul V, Hustvedt BE, Flanders WD (2008). Reliability and validity of self-reported physical activity in the Nord-Trondelag Health Study - HUNT 1. Scand J Publ Health.

[B13] Warburton DER, Nicol CW, Bredin SD (2006). Health benefits of physical activity: the evidence. Can Med Assoc J.

[B14] Kurtze N, Gundersen KT, Holmen J (2003). Self-reported physical activity in population studies - a methodological problem. Nor J Epidemiol.

[B15] King A, Wold B, Tudor-Smith C, Harel Y (1996). The health of youth. A cross national study..

[B16] Booth ML, Okely AD, Chey T, Bauman A (2001). The reliability and validity of the physical activity questions in the WHO health behaviour in schoolchildren (HBSC) survey: a population study. Br J Sports Med.

[B17] Craig CL, Marshall AL, Sjostrom M, Bauman AE, Booth ML, Ainsworth BE, Pratt M, Ekelund U, Yngve A, Sallis JF, Oja P (2003). International physical activity questionnaire: 12-country reliability and validity. Med Sci Sports Exerc.

[B18] (2005). Guidelines for the data processing and analysis of the
International Physical Activity Questionnaire. http://www.ipaq.ki.se/ipaq.htm.

[B19] Fogelholm M, Malmberg J, Suni J, Santtila M, Kyrolainen H, Mantysaari M, Oja P (2006). International physical activity questionnaire: Validity against fitness. Med Sci Sports Exerc.

[B20] Maddison R, Ni Mhurchu C, Jiang Y, Vander Hoorn S, Rodgers A, Lawes CM, Rush E (2007). International Physical Activity Questionnaire (IPAQ) and New Zealand Physical Activity Questionnaire (NZPAQ): A doubly labelled water validation.. Int J Behav Nutr Phys Act.

[B21] Hazzaa MAH (2007). Health-enhancing physical activity among Saudi adults using the International Physical Activity Questionnaire (IPAQ). Public Health Nutrition.

[B22] Holmen TL, Barret-Connor E, Clausen J, Langhammer A, Holmen JB (2002). Gender differences in the impact of adolescent smoking on lung function and respiratory symptoms. The Nord-Trøndelag Health study, Norway, 1995-1997. Respir Med.

[B23] Medbo JI, Mamen A, Welde B, von Heimburg E, Stokke R (2002). Examination of the Metamax I and II oxygen analysers during exercise studies in the laboratory. Scand J Clin Lab Invest.

[B24] Hustvedt BE, Christophersen A, Johnsen LR, Tomten H, McNeill G, Haggarty PL (2004). Description and validation of the ActiReg®: a novel instrument to measure physical activity and energy expenditure. Br J Nutr.

[B25] Andersen LF, Pollestad ML, Jacobs DR, Lovo A, Hustvedt BE (2005). Validation of a pre-coded food diary used among 13-year-olds: comparison of energy intake with energy expenditure. Public Health Nutrition.

[B26] Fredriksen PM, Ingjer F, Nystad W, Thaulow E (1998). Aerobic endurance testing of children and adolescents - a comparison of two treadmill protocols. Scand J Med Sci Sports.

[B27] Mifflin MD, Stjeor ST, Hill LA, Scott BJ, Daugherty SA, Koh YO (1990). A new predictive equation for resting energy-expenditure in healthy-individuals. Am J Clin Nutr.

[B28] Ainsworth BE, Haskell WL, Leon AS, Jacobs DR, Montoye HJ, Sallis JF, Paffenbarger RS (1993). Compendium of physical activities - classification of energy costs of human physical activities. Med Sci Sports Exerc.

[B29] Food and Agriculture Organization/World Health Organization(WHO)/United Nations University (1985). Energy and protein requirements. Report of a joint Expert Consultation. Geneva.

[B30] Landis JR, Koch GG (1977). The measurement of observer agreement for categorical data. Biometrics.

[B31] Treuth MS, Hou NQ, Young DR, Maynard LM (2005). Validity and reliability of the Fels Physical Activity Questionnaire for children. Med Sci Sports Exerc.

[B32] Vanhees L, Lefevre J, Philippaerts R, Martens M, Huygens W, Troosters T, Beunen G (2005). How to assess physical activity? How to assess physical fitness?. Eur J Cardiovasc Prev Rehab.

[B33] Ainsworth BE, Bassett DR, Strath SJ, Swartz AM, O'Brien WL, Thompson RW, Jones DA, Macera CA, Kimsey CD (2000). Comparison of three methods for measuring the time spent in physical activity. Med Sci Sports Exerc.

[B34] Macfarlane DJ, Lee CCY, Ho EYK, Chan KL, Chan D (2006). Convergent validity of six methods to assess physical activity in daily life. J Appl Physiol.

[B35] Eisenmann JC, Strath SJ, Shadrick D, Rigsby P, Hirsch N, Jacobson L (2004). Validity of uniaxial accelerometry during activities of daily living in children. Eur J Appl Physiol.

[B36] Arvidsson D, Slinde F, Nordenson A, Larsson S, Hulthen L (2006). Validity of the ActiReg system in assessing energy requirement in chronic obstructive pulmonary disease patients. Clin Nutr.

[B37] Myers J, Prakash M, Froelicher V, Do D, Partington S, Atwood JE (2002). Exercise capacity and mortality among men referred for exercise testing. N Engl J Med.

[B38] Shephard RJ, Vuillemin A (2003). Limits to the measurement of habitual physical activity by questionnaires. Br J Sports Med.

[B39] Fletcher GF, Balady GJ, Amsterdam EA, Chaitman B, Eckel R, Fleg J, Froelicher VF, Leon AS, Pina IL, Rodney R, Simons-Morton DG, Williams MA, Bazzarre T (2001). Exercise standards for testing and training - A statement for healthcare professionals from the American Heart Association. Circulation.

[B40] Rognmo O, Hetland E, Helgerud J, Hoff J, Slordahl SA (2004). High intensity aerobic interval exercise is superior to moderate intensity exercise for increasing aerobic capacity in patients with coronary artery disease. Eur J Cardiovasc Prev Rehab.

[B41] Mcmurray RG, Ring KB, Treuth MS, Welk GJ, Pate RR, Schmitz KH, Pickrel JL, Gonzalez V, Jaoa M, Almedia CA, Young DR, Sallis JF (2004). Comparison of two approaches to structured physical activity surveys for adolescents. Med Sci Sports Exerc.

[B42] Florindo AA, Romero A, Peres SV, da Silva MV, Slater B (2006). Development and validation of a physical activity assessment questionnaire for adolescents. Rev Saude Publica.

[B43] Ainslie PN, Reilly T, Westerterp KR (2003). Estimating human energy expenditure - A review of techniques with particular reference to doubly labelled water. Sports Med.

[B44] Pettersen SA, Fredriksen PM, Ingjer F (2001). The correlation between peak oxygen uptake (VO2peak) and running performance in children and adolescents. Aspects of different units. Scand J Med Sci in Sports.

[B45] Whitlock EP, Williams SB, Gold R, Smith PR, Shipman SA (2005). Screening and interventions for childhood overweight: A summary of evidence for the US preventive services task force. Pediatrics.

